# High-resolution cryo-EM structures of the *E*. *coli* hemolysin ClyA oligomers

**DOI:** 10.1371/journal.pone.0213423

**Published:** 2019-05-02

**Authors:** Wei Peng, Marcela de Souza Santos, Yang Li, Diana R. Tomchick, Kim Orth

**Affiliations:** 1 Department of Molecular Biology, University of Texas Southwestern Medical Center, Dallas, TX, United States of America; 2 Howard Hughes Medical Institute, University of Texas Southwestern Medical Center, Dallas, TX, United States of America; 3 Department of Biophysics, University of Texas Southwestern Medical Center, Dallas, TX, United States of America; 4 Department of Biochemistry, University of Texas Southwestern Medical Center, Dallas, TX, United States of America; East Carolina University Brody School of Medicine, UNITED STATES

## Abstract

Pore-forming proteins (PFPs) represent a functionally important protein family, that are found in organisms from viruses to humans. As a major branch of PFPs, bacteria pore-forming toxins (PFTs) permeabilize membranes and usually cause the death of target cells. *E*. *coli* hemolysin ClyA is the first member with the pore complex structure solved among α-PFTs, employing α-helices as transmembrane elements. ClyA is proposed to form pores composed of various numbers of protomers. With high-resolution cryo-EM structures, we observe that ClyA pore complexes can exist as newly confirmed oligomers of a tridecamer and a tetradecamer, at estimated resolutions of 3.2 Å and 4.3 Å, respectively. The 2.8 Å cryo-EM structure of a dodecamer dramatically improves the existing structural model. Structural analysis indicates that protomers from distinct oligomers resemble each other and neighboring protomers adopt a conserved interaction mode. We also show a stabilized intermediate state of ClyA during the transition process from soluble monomers to pore complexes. Unexpectedly, even without the formation of mature pore complexes, ClyA can permeabilize membranes and allow leakage of particles less than ~400 Daltons. In addition, we are the first to show that ClyA forms pore complexes in the presence of cholesterol within artificial liposomes. These findings provide new mechanistic insights into the dynamic process of pore assembly for the prototypical α-PFT ClyA.

## Introduction

As the largest family of bacterial toxins, pore-forming toxins (PFTs) represent important factors for bacterial virulence. The protein family is characterized by the pore-forming activity and these types of proteins exist in other kingdoms of life [1, 2]. Proteins exhibiting pore-forming activity, named as pore-forming proteins (PFPs), cause permeation of the target membrane, disruption of cellular homeostasis and death of target cells. With respect to protein structure, PFTs can be divided into two classes, α-PFTs, and β-PFTs, depending on the secondary structure of transmembrane elements of α-helices and β-barrels, respectively [1, 2].

*E*. *coli* cytolysin A (ClyA), also known as hemolysin (HlyE) or silent hemolysin A (SheA), belongs to the α-PFT class [2, 3]. ClyA has been shown to be responsible for the hemolytic activity of certain strains of *E*. *coli*, including the pathogenic strain *E*. *coli* O157 [4–6]. Homologs of ClyA are also found in other pathogenic bacteria, including *Salmonella typhi* and *Shigella flexneri* [5, 7]. Distinct from many secreted toxins and effectors, ClyA is observed to be delivered by outer-membrane vesicles (OMVs) [8]. As the first α-PFT with the structure of a pore complex determined, ClyA represents the prototype in the ClyA α-PFT subfamily [3]. Other subfamily members include NheA and Hbl-B from *Bacillus cereus*, and Cry6Aa from Bacillus thuringiensis, which share obvious sequence or structural similarities with ClyA [9–11]. ClyA is a 34 kDa soluble monomer, containing mainly α-helices with only one β-tongue that transforms into α-helical elements in the pore complexes [3, 12].

Various low-resolution EM analyses suggest that the ClyA pore complexes form 6-, 8- or 13-fold symmetrical structures [13, 14]. However, the crystal structure of ClyA pore complex displays only a 12-meric composition [3]. Due to the limitations of the protein crystal lattice, which self-selects for a single oligomeric species, it is reasonable to propose the existence of ClyA pore complexes of different oligomeric states. Here we report the cryo-EM structures of ClyA pore complexes in the presence of n-Dodecyl-β-D-Maltoside (DDM) as dodecamer, tridecamer, and tetradecamer at the resolution of 2.8 Å, 3.2 Å and 4.3 Å, respectively. These structures clearly reveal the diverse organizations of ClyA pore complexes, with high-resolution electron microscopy density maps contributing to more accurate assignments of amino acid residues for the dodecamer. Structure comparisons indicate a rigidity of protomers when assembled into pore complexes. We also show that a mild detergent, digitonin, is able to facilitate the transition of ClyA from soluble monomer to an intermediate state. With the presence of cholesterol within artificial liposomes, ClyA forms pore complexes. Overall, our studies reveal the pore assembly mechanism involving intermediate oligomers for the formation of a prototypical α-PFT of ClyA with varying numbers of protomers.

## Results

### Assembly of the ClyA pore complexes

Detergents, including DDM and n-Octyl-β-D-Glucopyranoside (β-OG), have been shown to induce the *in vitro* formation of the ClyA pore complexes [3, 13, 14]. We first tested the effect of DDM on the assembly of the ClyA complexes. As shown in **S1A Fig**, DDM caused a dramatic shift of the ClyA protein elution peak to a smaller elution volume with size exclusion chromatography (SEC), indicating the formation of large oligomers of ClyA pore complexes. Characteristic ClyA pore complexes were observed with negative staining EM images (**S1B Fig**). Interestingly, the ClyA pore complexes from an earlier or later eluted fraction with SEC exhibited slightly bigger or smaller central pore size, respectively, implying a heterogeneous composition of ClyA pore complexes. The variance of ClyA pore size was also observed in cryo-EM images (**S4A Fig**). Soluble ClyA monomer was previously found to assemble into a 13-meric pore complex in the presence of DDM [14], but a dodecamer was observed in the crystal structure [3]. EM images of ClyA pore complexes from a β-OG/lipid mixture displayed 6- or 8-fold symmetry [13]. To determine the oligomerization states of the complexes we obtained, we took advantage of cryo-EM for further investigation.

### Structure determination of the ClyA pore complexes

We pooled all fractions containing ClyA pore complexes in DDM after SEC for cryo-EM grid preparation. With this sample numbered as sample #1 (**S1C Fig**), we were able to perform grid screening and collect micrographs on a Talos Arctica operated at 200 kV. After data processing, we successfully obtained characteristic 2D class averages, with a relatively small dataset of 410 micrographs containing 22,998 auto-picked particles (**S2A** and **S2C Fig**). Two top view images (or bottom view, as they cannot be differentiated) clearly indicated the presence of both dodecamer and tridecamer (**S2A Fig**). 3D reconstruction with further classified particles produced low-resolution density maps of the dodecamer and the tridecamer, at resolutions of 6.8 Å and 7.5 Å, respectively. Based on the particle numbers of total particles and those used in final 3D reconstructions, the majority of the complexes were 12-meric, representing approximately 80% of all particles, while only 11% were 13-meric. These observations confirm the existence of 13-metric ClyA pore complexes [14], albeit at a low concentration in this preparation.

Digitonin is generally considered to be a mild detergent and has been used successfully for cryo-EM structure determinations of membrane proteins [15–17]. Therefore, we replaced DDM with digitonin after pore formation and added an additional step of Ni^2+^-NTA affinity purification before SEC (sample #2, **S1C Fig**). The data from sample #2 obtained on the same microscope (Talos Arctica) yielded a dodecamer density map at the resolution of 3.9 Å from fewer particles (**S2D Fig**). The tridecamer was lost during this sample preparation process, as evidenced by the lack of 2D class averages for a tridecamer (**S2B** and **S2D Fig**). In the hopes to visualize the tridecamer, we subsequently adopted a purification protocol by skipping the Ni^2+^-NTA affinity purification and directly applying the DDM complex to SEC with digitonin in the buffer (sample #3, **S1C Fig**). Data for sample #3 were collected on a Titan Krios (300 kV) to obtain micrographs of better quality.

**Fig 1:**
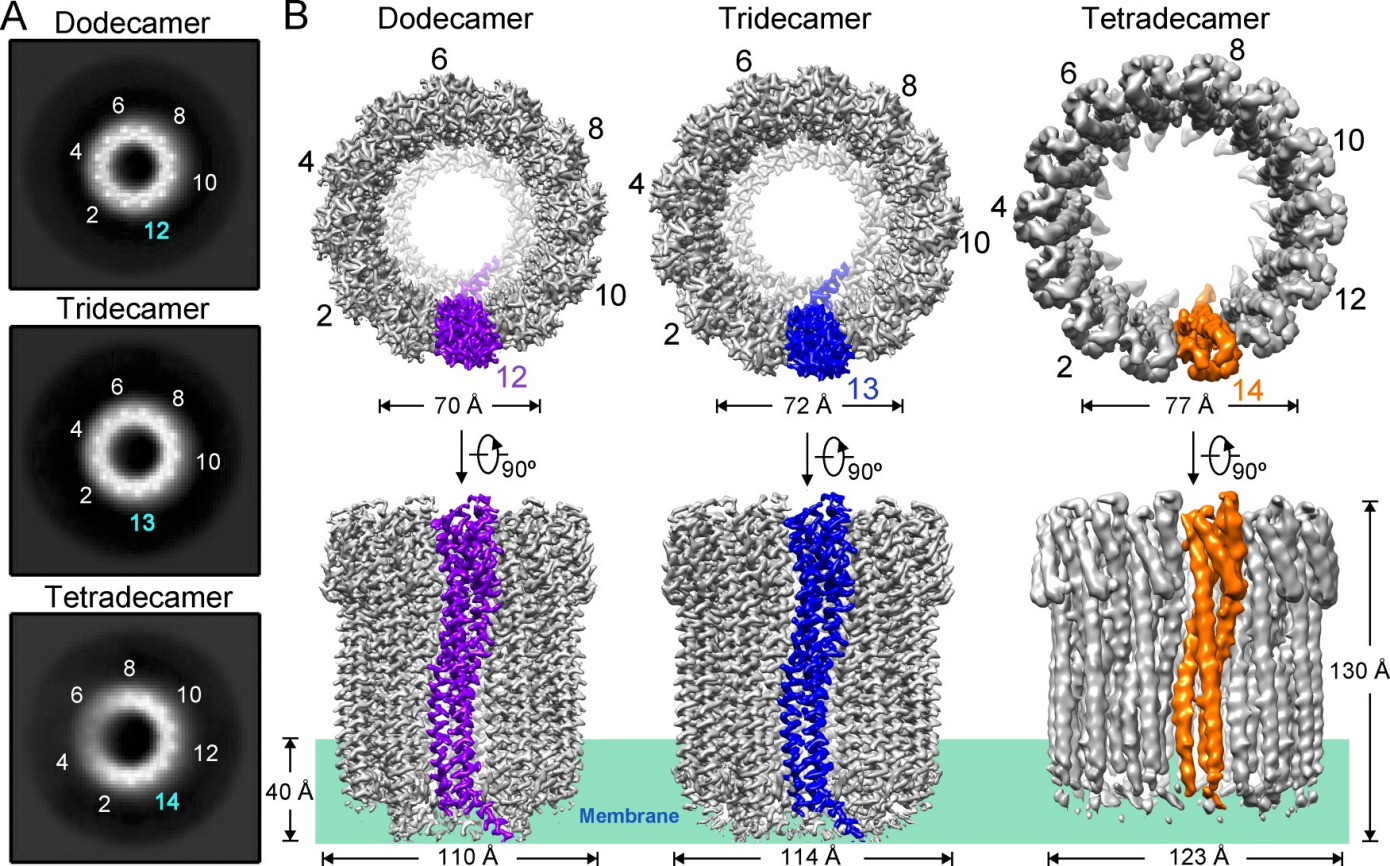
Cryo-EM structures of the ClyA pore complexes. (**A**) Top view (or bottom view) of the dodecamer, the tridecamer and the tetradecamer from 2D class averages of the ClyA pore complexes. The numbers of symmetric features are indicated. (**B**) The final three-dimensional reconstruction electron microscopy density maps of the dodecamer, the tridecamer, and the tetradecamer at the resolution of 2.8 Å, 3.2 Å and 4.3 Å. Top views and side views are shown. One protomer from each oligomer is shown in purple, blue or orange for clarity.

With the data from sample #3, we were able to observe not only the tridecamer in addition to the dodecamer but also a new oligomer with 14-fold symmetry (**Figs 1A**, **S3** and **S4B**). Final 3D reconstruction resulted in EM density maps of the dodecamer, the tridecamer and the tetradecamer at the resolution of 2.8 Å, 3.2 Å and 4.3 Å, respectively (**Figs 1B**, **S3** and **S4**; **S1 Table**). The three density maps clearly exhibit distinct secondary structural elements, demonstrating that DDM is capable of inducing the formation of ClyA pore complexes of multiple oligomeric states. The near-atomic resolution maps of the dodecamer and the tridecamer allowed reasonable model building with accurate side chain assignments. Although the tetradecamer map was resolved at a relatively low resolution, the model was still built based on the dodecamer structure model with identical protomer fold and inspection of the electron microscopy density maps for assignment of bulky residues (**Figs 1B** and **S5**; **S1 Table**).

### Structure comparison of the ClyA pore complexes

**Fig 2:**
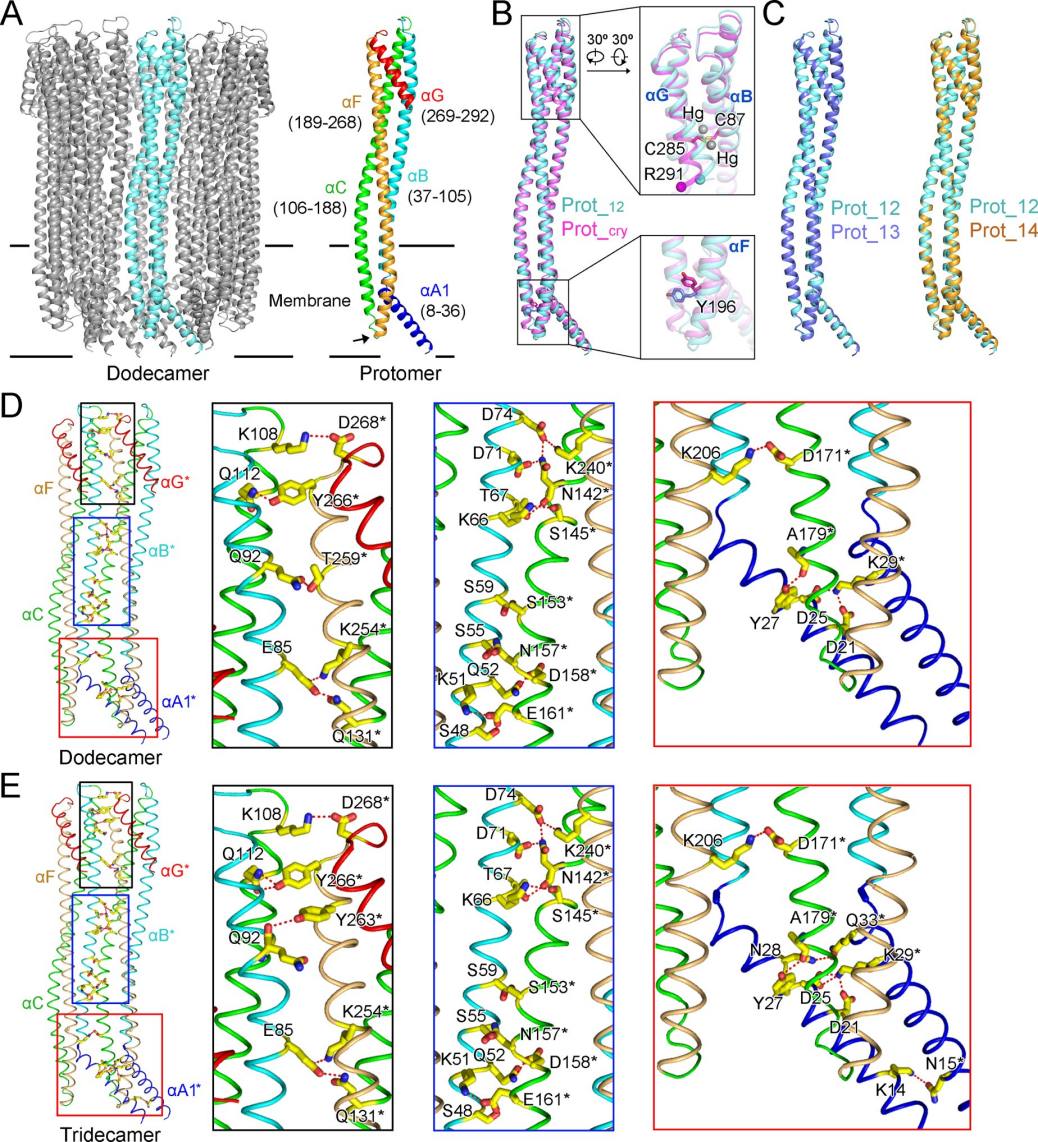
Structure comparison of the ClyA dodecamer, tridecamer, and tetradecamer complexes. (**A**) Illustration of secondary elements within one protomer from the dodecamer. Five α-helices (αA1, αB, αC, αF, and αG) within one protomer (left) are colored in blue, cyan, green, orange and red (right). The corresponding amino acid boundaries are indicated, with the short loops divided and assigned to the linked helices for simplicity. The turning point of αC and αF is indicated by an arrow. The same coloring paradigm is applied in **Figs 2D**, **2E** and **S7**. (**B**) Comparison of one protomer from the dodecamer (Prot_12, in cyan) with that from the crystal structure (Prot_cry, in magenta). The PDB accession code for the crystal structure is 2WCD. The two mercury atoms in the crystal structure are shown as grey balls. Cα atoms of R291 are indicated as balls. (**C**) Comparison of Prot_12 with a protomer from the tridecamer (Prot_13, in blue) and the tetradecamer (Prot_14, in orange). (**D**, **E**) Extensive hydrogen bonds and salt bridges mediate the interaction between neighboring protomers in the dodecamer and the tridecamer. Residues involved are labeled and shown as sticks. Residues from different protomers are distinguished with or without “*”.

For membrane proteins, the transmembrane regions are usually more stable, and the local resolutions are better compared to those of the soluble regions. However, this is not the case for the ClyA pore complexes, since the local resolutions of all three transmembrane regions are worse than that of the soluble regions (**S4E Fig**). This may result from the incomplete transmembrane helices from αC and αF, with the turning point located within rather than outside of the membrane, making the transmembrane regions unstable (**Fig 2A**, black arrow). Although the N-terminus of αA1 helix from the tetradecamer is even more disordered and not well resolved, we built the model similar to the dodecamer (**Figs 1B**, **S4E** and **S5**).

The height of the dodecamer, the tridecamer or the tetradecamer is approximately 130 Å. The outer diameter varies from 110 Å to 123 Å as ClyA pore complexes incorporate more protomers. The inner diameters near the extracellular edge of the pore are 70, 72 and 77 Å for the dodecamer, the tridecamer, and the tetradecamer, respectively. The narrowest part along the pore is located at the membrane, where the inner diameters vary from 40 Å to 52 Å (**Figs 1B** and **S6**).

Each protomer in the ClyA pore complexes is composed of five α-helices, namely αA1, αB, αC, αF, and αG (**Fig 2A**). The crystal structure of the ClyA pore complex was previously solved as a dodecamer at 3.3 Å [3]. The EM density map of the dodecamer presented here is at 2.8 Å, representing an improved density and structure model (**S5 Fig**). One typical example is the side chain of Y196 from αF, which is buried in the membrane. With the cryo-EM density map, we were able to re-assign the side chain in parallel to, rather than pointing toward the hydrophobic membrane environment (**Figs 2B** and **S5**). Sodium ethylmercurithiosalicylate was used as the heavy atom source for structure determination of the crystal structure, and two mercury atoms are bound to the only two cysteine residues; C87 from αB and C285 from αG [3]. One protomer from the dodecamer (Prot_12) was superimposed to a protomer from the crystal structure (Prot_cry) (**Fig 2B**). Based on the comparison, it is obvious that the binding of mercury atoms pushes away the C-terminus of the αG helix with a movement distance of 4.6 Å at the Cα of R291 (**Fig 2B**). This represents an artificial distortion of the local structure by heavy atoms, although the overall fold is not affected [3].

Comparison between Prot_12 and a protomer from the tridecamer (Prot_13) or the tetradecamer (Prot_14) shows that the overall fold of the protomer does not change when forming various oligomers, indicating a rigid body protomer assembly mechanism for the ClyA pore complexes (**Fig 2C**). Extensive hydrogen bonds and salt bridges, spanning from the transmembrane regions to the distal soluble regions, mediate the interaction between neighboring protomers within the dodecamer, which is also observed in the crystal structure (**Figs 2D** and **S7**) [3]. The interaction paradigm remains highly conserved in the tridecamer, suggesting a single protomer-protomer interaction mode in the ClyA pore complexes (**Fig 2E**).

### An Intermediate state of ClyA during transition into pore complexes

**Fig 3:**
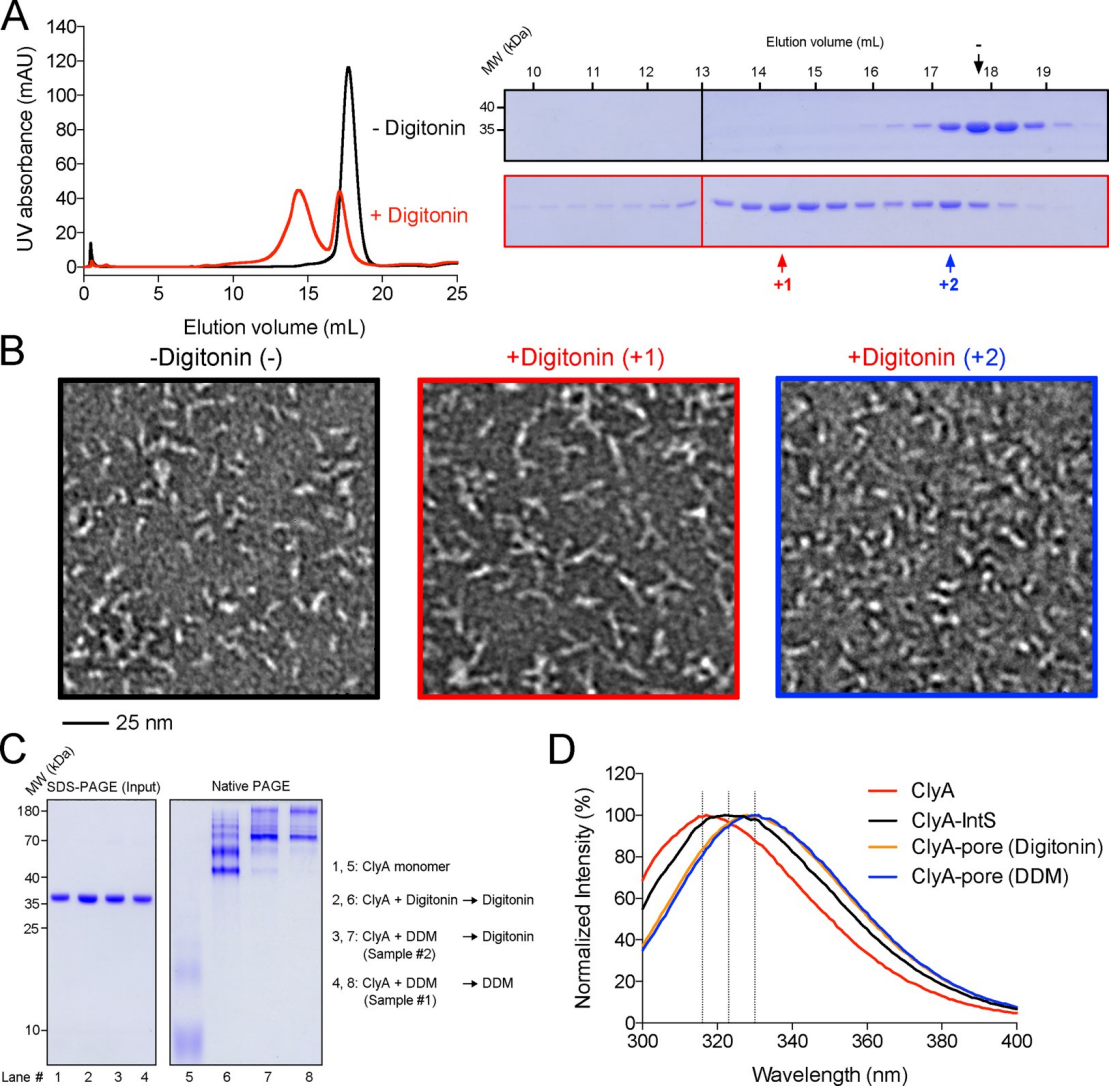
Characterization of a ClyA intermediate state. (**A**) SEC profile of ClyA in the soluble state and an intermediate state. Digitonin (1%) was incubated with ClyA overnight before gel filtration to obtain the intermediate state sample. The elution volumes for the two states were 17.7 mL and 14.4 mL (Superose 6 10/300 GL, GE Healthcare), respectively. (**B**) Negative staining images of ClyA in the form of soluble state and intermediate state. Fractions indicated in (**A**) were applied to negative staining (“-” indicates the soluble protein, “+1” or “+2” indicates the sample with digitonin). (**C**) Native PAGE analysis of ClyA in the soluble state (lane 1, 5), the intermediate state (lane 2, 6), and the state of mature pore complex (lane 3, 4, 7, 8). (**D**) Fluorescence emission spectra of the ClyA monomer, the intermediate state (IntS) and the pore complexes. Wavelengths of 316, 323, and 330 nm are indicated for clear visualization of peak shift.

As discussed above, the detergent digitonin was helpful for determining the high-resolution cryo-EM structures of ClyA pore complexes. However, DDM was still necessary for the formation of mature pore complexes before its replacement with digitonin, as incubation of soluble ClyA monomers with digitonin did not promote the formation of ClyA pore complexes (**Fig 3A** and **3B**). Unexpectedly, ClyA incubated with digitonin formed trimer-like complexes (**Fig 3B**). Native PAGE gel confirmed the medium size of the trimer-like complex between the soluble monomer and the mature pore complex (**Fig 3C**). The digitonin-induced trimer probably represents an intermediate state of ClyA during the transition from soluble monomers to mature pore complexes. The fluorescence emission spectra of the intermediate state differ from the soluble monomer and pore complex, suggesting a different conformation and further supporting that the digitonin induced complex represents an intermediate.

To further characterize the ClyA trimer, we also collected cryo-EM micrographs on a Talos Arctica. More than 15,000 particles were picked from the images. After data processing, several 2D class averages confirmed the trimeric feature of the intermediate state, though others showed tetrameric feature (**S8A Fig**). 3D classification and reconstruction with approximately 5,000 classified particles resulted in an EM density map at the resolution of 16 Å (**S8B Fig**). With this low-resolution map, details on the protomer structure and the interaction between protomers could not be discerned. However, based on the overall long-rod shape of the ClyA monomer and the protomer (**S8B** and **S8C Fig**), the trimer organization manner must be either head-to-head or tail-to-tail.

The transition of ClyA from soluble monomers to pore complexes involves significant conformational changes of αA1, αA2, αD, β-tongue, and αE, which insert into the membrane, while αB, αC, αF and αG function as the scaffold (**Figs 2A** and **S8C**) [3, 12]. Partial scaffold elements could self-assemble into ring-like structures without membrane-buried elements [13], explained by the extensive protomer-protomer interactions (**Fig 2D** and **2E**). Therefore, the corresponding transmembrane elements in the soluble monomer prior to complete rearrangement in the protomer inhibit the assembly of ClyA ring-like pore complex. The intermediate state shown here appears as a trimer, indicating that digitonin is not able to completely disrupt the interactions stabilizing ClyA as a monomer and facilitate the maturation of the ClyA pore complexes.

### The interaction between ClyA and liposomes

ClyA is capable of forming pores on artificial liposomes made of porcine brain total lipid extract (BTLE) [12–14]. Therefore, we carried out a liposome leakage assay for testing the interaction of ClyA with membrane lipids and its ability to permeabilize liposomes. Liposomes were prepared with 1-palmitoyl-2-oleoyl-glycero-3-phosphocholine (POPC) and 1,2-dioleoyl-sn-glycero-3-phospho-L-serine (DOPS) or BTLE and then tested for the leakage activity of ClyA. A *Vibrio parahaemolyticus* type III secretion system effector, VopQ (known to induce liposome leakage), and its chaperone VP1682, were tested as positive and negative controls, respectively, in the liposome leakage assay [18, 19]. At pH 5.5, VopQ causes fast and dramatic 5(6)-Carboxyfluorescein (CF) dye release from POPC-DOPS liposomes, while at pH 7.4, the leakage activity is less efficient (**S9A Fig**), consistent with previous observations [18]. Addition of ClyA showed similar leakage activity with POPC-DOPS liposomes (**S9A Fig**), with higher leakage activity at lower pH. The observations may be explained by changes in the protein overall charge, as the estimated pIs of VopQ and ClyA are 5.7 and 5.1, respectively [18]. Experiments done at a low pH caused the pore-forming proteins to be more positively charged, enabling them to bind more efficiently to the negatively charged liposomes. ClyA also caused leakage of BTLE liposomes, which contained more diverse mammalian cell lipids (**S9B Fig**). Pre-formed ClyA pore complexes were also tested and found to show no obvious leakage activity (**S9B Fig**). This is consistent with observations that mature ClyA pore complexes show little hemolytic activity on erythrocytes [20, 21].

**Fig 4:**
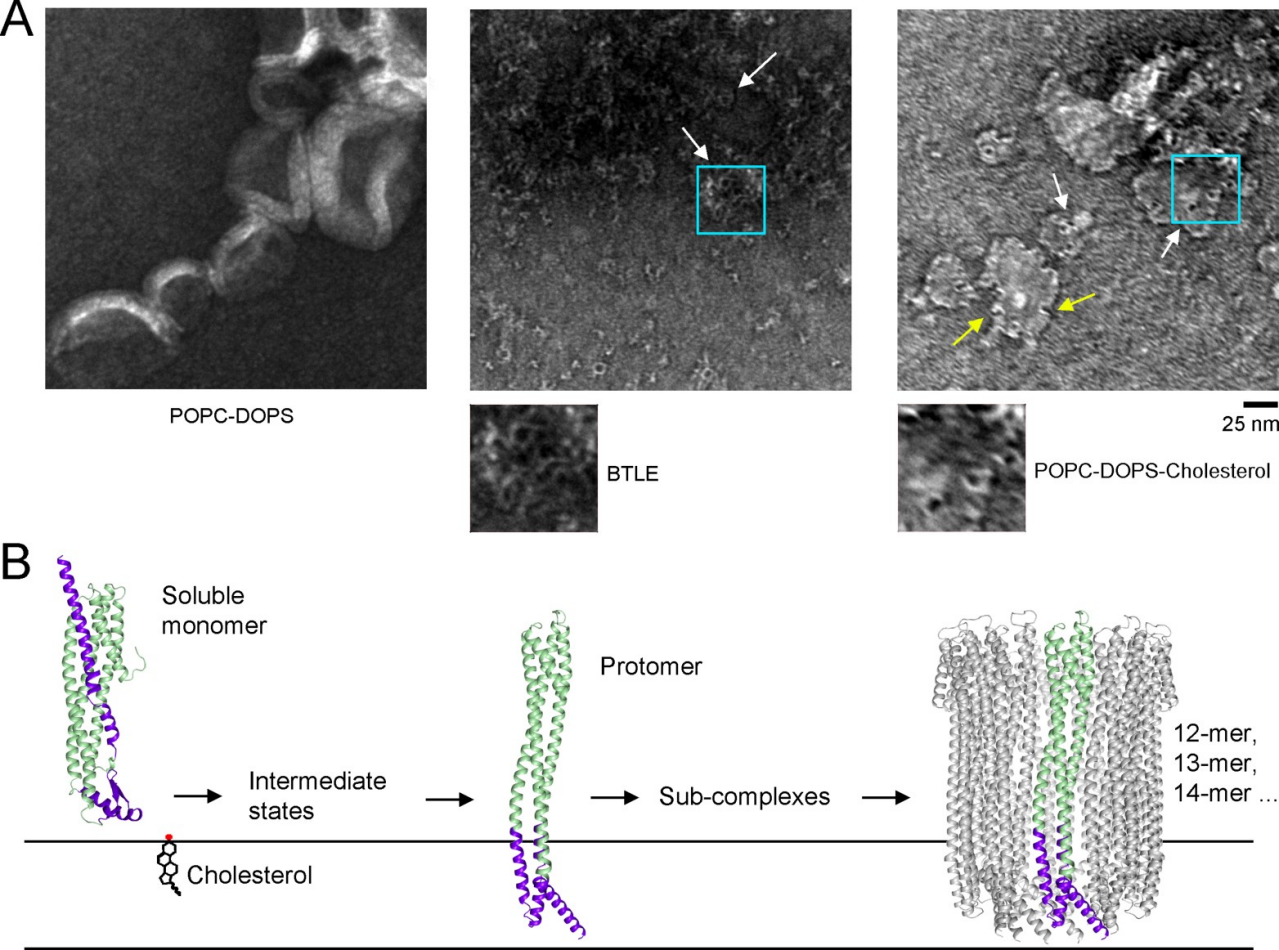
ClyA forms pores on cholesterol-containing liposomes. (**A**) POPC-DOPS, POPC-DOPS-cholesterol or BTLE liposomes were incubated ON with 5 μM ClyA (-tag) at 4°C (pH 7.4) and examined with negative staining. ClyA forms pore structures on POPC-DOPS-cholesterol and BTLE liposomes, but not on POPC-DOPS liposomes. Representative pores are indicated with white arrows. Regions within the cyan boxes are enlarged for clear visualization. Crescent structures are indicated with yellow arrows. (**B**) Model for the assembly of ClyA pore complexes from the soluble monomer. The corresponding transmembrane elements in the soluble monomer and one protomer are colored purple.

We examined whether ClyA formed visible pore complexes on the same liposomes used in our leakage assay by negative staining. As shown in **S9C Fig**, ClyA did not form obvious pore structures on POPC-DOPS liposomes, even though we observed dramatic leakage activity (**S9A Fig**). Since the protein tested contained an N-terminal affinity purification tag (21 amino acids), which would relocate from the outside of membrane to the inside and may impede pore complex formation, we removed the tag and tested ClyA (-tag). In the presence of DDM, the tag-free ClyA protein formed pores similar to those formed by the tagged protein, as expected, based on the fluorescence emission spectra (**S8D Fig**). Even at a higher concentration (5 μM), ClyA (-tag) still could not form pores on POPC-DOPS liposomes (**Fig 4A**). ClyA was previously shown to form pores on BTLE liposomes [12–14]. We then tested BTLE liposomes with different lipid composition and did find pore-like structures (**Fig 4A**). Cholesterol has been revealed to facilitate membrane binding and pore complex stabilization of ClyA [22, 23]. Cholesterol is not listed as a component on the manufacturer’s data sheet, but approximately 60% of BTLE ingredients are unknown. As a common animal membrane component, cholesterol would be expected to be present in the BTLE, explaining the observation that ClyA pores were found on BTLE liposomes. To test more carefully the effect of cholesterol, we prepared POPC-DOPS-cholesterol liposomes and indeed observed pore-like structures on these liposomes (**Fig 4A**).

The above observations on the liposome leakage activity and the pore formation on POPC-DOPS liposomes suggest that the leakage activity of a ClyA protein (monomer, intermediate state or protomer) is not equivalent to the membrane permeability mediated by mature pore complexes of ClyA. Therefore, the leakage activity on liposomes can be uncoupled from the pore-forming activity for ClyA, and possibly for any given protein with pore-forming activity. Moreover, the irrelevance of the leakage activity to the pore-forming activity also indicates that ClyA pore complexes are not essential for leakage of molecules smaller than the 376 Dalton CF dye. The initial interaction between ClyA monomer and membrane would thus cause membrane instability and leakage, even before the formation of mature ClyA pore complexes, which is suggested by the leakage kinetics of ClyA [24].

## Discussion

PFTs are important bacterial virulence factors with pore-forming activity on target membranes [1, 2]. As a prototypical α-PFT, ClyA from *E*. *coli* assembles into pore structures on the membrane with a pore size of 40 Å [3, 12]. There has been controversy about the organization of ClyA pore complexes with low-resolution EM analysis [3, 13, 14]. In addition to the dodecamer which has been characterized by protein crystallography [3], here we report high-resolution cryo-EM structures of the tridecamer and the tetradecamer from a single dataset. The density maps allow accurate structural model building and elucidation of [25] the profile of ClyA pore complexes. Based on the cryo-EM structures, we eliminated artifacts such as the movement of the αG helix due to mercury atoms bound in the crystal structure [3]. The pore complex protomers from the tridecamer and the tetradecamer exhibit the same fold as observed with the dodecamer, indicating the rigidity of ClyA protomer when assembled into pore complexes. The protomer-protomer interaction paradigm in the dodecamer applies also to the tridecamer, and probably the tetradecamer.

Various oligomerization states for PFTs or PFPs assembly have also recently been observed via cryo-EM single particle analysis, for a two-component α-PFT YaxAB from *Yersinia enterocolitica*, or XaxAB from *Xenorhabdus nematophila*, and a pyroptotic β-PFP of gasdermin A3 [25–27]. This illustrates the advantage of cryo-EM in determining distinct organization states of PFP complexes, which could not be achieved with protein crystallography [3]. The distinct organization states for PFPs are not surprising due to the flexibility or plasticity of high-order assembly, which may or may not possess functional difference.

Outer membrane vesicles (OMVs) are used for export, delivery and, activation of ClyA *in vivo* [8]. However, ClyA pores on OMVs seemed to exhibit an inverted tail-outside orientation. This orientation is particularly intriguing because it requires shuttling the large ClyA tail–rather than the comparatively small head group–across the vesicular membrane [8]. The tail-outside orientation of ClyA was observed on BTLE liposomes, which is to be expected, as the ClyA protein was added from outside (data not shown) [12–14]. Unlike on BTLE liposomes, ClyA monomer could not assemble into pore complexes on liposomes prepared with *E*. *coli* total lipid extract [21]. An oligomeric pre-pore state may be escorted by OMVs, representing a possible mechanism for ClyA delivery [21]. In vitro, ClyA is shown to form assembly-competent protomer, elongate as sub-complexes and, finally, assemble as mature pore complexes [28]. As a support for this mechanism, crescent sub-complexes are observed on POPC-DOPS-cholesterol liposomes (**Fig 4A**). Thus, the *in vivo* delivery and assembly mechanism of ClyA still requires further investigation.

Treatment with the mild detergent digitonin is able to induce the formation of a putative intermediate state of ClyA and stabilize the state for long time range study. The cryo-EM analysis results imply that the trimer interaction interface is mediated by the heads or the tails. However, due to the low resolution, detailed structural information about the intermediate state could not be addressed yet. Future investigation on intermediate states may benefit from more detergent options and other biophysical methods, as seen with FRET [28]. Both mature pores and ring-like pre-pores with immature transmembrane elements (non-membrane-inserted) have been observed for β-PFPs, e.g., α-hemolysin and bi-component γ-hemolysin from *Staphylococcus aureus* and gasdermin A3 [27, 29–31]. However, no pre-pore structure has been identified for α-PFPs, even though a high-order oligomer is observed for YaxAB and multiple states are revealed for FraC from *Actinia fragacea* [26, 32]. Further investigations on the pre-pore structures of α-PFPs are necessary for the complete illustration of α-PFP assembly mechanism.

ClyA is capable of inducing leakage of liposomes with a simple composition of POPC and DOPS. Cholesterol has been revealed to facilitate membrane binding and pore complex stabilization [22, 23], which may explain the pore formation of ClyA on BTLE liposomes but not POPC-DOPS liposomes. By separating the liposome leakage activity from the pore-forming activity, we demonstrate that binding of ClyA is sufficient to cause liposome leakage of small molecules without the formation of mature pores on liposome membranes. This may also be applicable to other PFPs, adding caution to the interpretation of liposome leakage assay data.

Overall, we have demonstrated that ClyA can form pores of various sizes, including a dodecamer, tridecamer, and tetradecamer. We show an intermediate state of ClyA that is induced and stabilized by digitonin. The ClyA monomer, before assembling into pore complexes, can permeabilize membranes and allow leakage of particles less than ~400 Daltons. Cholesterol facilitates the assembly of the ClyA pore complexes on membranes. These findings provide new insights into the assembly mechanism for ClyA pore complexes (**Fig 4B**).

## Materials and methods

### Expression and purification of ClyA

The cDNA encoding *E*. *coli* ClyA was initially amplified from BL21 (DE3). Mutations and deletions were removed by two-step PCR. The corrected fragment was cloned into the first multiple cloning site of a modified pETDuet-1 vector, with an N-terminal 6×His tag followed by a DrICE cutting site (DEVDA). The final plasmid was sequenced and contained a mutation (A248V) compared to the sequence deposited in UniProtKB (accession code: P77335). The plasmid was transformed into BL21 (DE3) strain for protein over-expression. When OD_600_ reached 1.0, the temperature was adjusted to 16°C, and 0.2 mM IPTG was added for induction overnight. Cells were collected by centrifugation and re-suspended in lysis buffer (25 mM Tris-HCl, pH 8.0, and 150 mM NaCl). After removal of cell debris by centrifugation at 22,000 g for 1 h, the supernatant was loaded to Ni^2+^-NTA resin (Qiagen). The resin was washed by buffer W1 containing 25 mM Tris-HCl, pH 8.0, and 300 mM NaCl, and sequentially by buffer W2 containing 25 mM Tris-HCl, pH 8.0, and 10 mM imidazole. Protein bound to the resin was eluted by buffer E containing 25 mM Tris-HCl, pH 8.0, and 300 mM imidazole. Anion exchange chromatography (Mono Q 5/50 GL, GE Healthcare) was performed for further purification. Coomassie Plus Protein Assay Reagent (Thermo Fisher Scientific) was used for protein concentration determination. The protein was flash-frozen by liquid nitrogen and stored at -80°C for later use. Unless noted, ClyA with complete purification tag (MGSSHHHHHHSQDLDEVDAGS-) was used. For tag removal to produce ClyA (-tag), tagged ClyA was treated with DrICE and applied to gel filtration for further purification.

### Assembly of the ClyA pore complexes

The ClyA pore complexes were similarly obtained using detergent n-dodecyl-β-D-maltoside (DDM) according to a previous protocol [14]. Briefly, ClyA protein (2.5 mg/mL) was incubated with 0.5% DDM in the presence of 5 mM DTT at 4°C overnight. The induced pore complexes were applied to size exclusion chromatography (SEC) with a column of Superdex 200 10/300 GL (GE Healthcare) in the buffer containing 25 mM Tris-HCl, pH 8.0, 150 mM NaCl, 0.05% Digitonin and 2 mM DTT. Fractions containing ClyA pore complexes were pooled together and concentrated to approximately 12 mg/mL for cryo-EM grid preparation.

### Negative staining sample preparation

3.5 μL of the protein or liposome sample was applied onto a glow-discharged grid (CF400-Cu, Electron Microscopy Sciences) and placed at RT for 1 min. The sample was then removed with filter paper and the grid was washed 3 times by 2% Uranyl acetate drop. Negative staining images were obtained on an FEI Tecnai G2 Spirit Biotwin microscope operated at 120 kV.

### Cryo-EM image collection

Cryo-EM grids were prepared using Vitrobot Mark IV (Thermo Fisher Scientific). 3.5 μL of protein was placed on a glow-discharged Quantifoil R1.2/1.3 300-mesh gold holey carbon grid. The grid was immediately blotted for 3.5 s under 100% humidity at 8°C before being plunged into liquid ethane. For grid screening, micrographs were acquired on a Talos Arctica microscope (Thermo Fisher Scientific) operated at 200 kV with a K2 Summit direct electron detector (Gatan). Images were recorded with SerialEM software [33]. For high-quality data collection, micrographs were acquired on a Titan Krios microscope (Thermo Fisher Scientific) operated at 300 kV with a K2 Summit direct electron detector, using a slit width of 20 eV on a GIF-Quantum energy filter. Images were recorded with EPU software (Thermo Fisher Scientific) at the calibrated magnification of 46,730× in counting mode with a super-resolution pixel size of 0.535 Å. The defocus range was set from -1.6 μm to -2.6 μm. Each micrograph was dose-fractionated to 30 frames under a dose rate of 4 e^-^/pixel/s, with a total exposure time of 15 s resulting in a total dose of around 50 e^-^/Å^2^.

### Cryo-EM image processing

Image processing was roughly done following the RELION 2.1 user manual [34, 35]. Relion 2.1 was compiled by SBGrid [36]. Data obtained on Talos Arctica were processed similarly as those obtained on Titan Krios. The Titan Krios micrographs were motion corrected and binned two-fold with MotionCor2 [37], yielding a pixel size of 1.07 Å. The CTF parameters of the micrographs were estimated using CTFFIND 4 [38]. All other steps of image processing were performed using RELION 2.1 [34, 35]. Around 1,000 particles were manually picked from a few micrographs for initial 2D classification and template generation for auto-picking. 737,817 particles were then auto-picked from 2,820 selected micrographs. After 2 rounds of 2D classification, a total of 667,297 particles were selected for further processing. An initial refinement with these particles resulted in an electron microscopy density map with C12 symmetry of 2.88 Å. 3D classification with C12, C13 or C14 symmetry allowed the separation of particles with distinct symmetries. 482,946 particles with C12 symmetry, 68,997 particles with C13 symmetry or 30,904 particles with C14 symmetry, were selected for the dodecamer, the tridecamer or the tetradecamer 3D reconstruction. Particle polishing was applied to the dodecamer and the tridecamer 3D reconstruction. An optimized soft mask was finally generated and applied to each oligomer. This resulted in the electron microscopy density maps of the dodecamer, the tridecamer, and the tetradecamer at 2.80 Å, 3.19 Å and 4.34 Å, respectively. The resolutions were estimated according to the gold-standard Fourier shell correlation (FSC) 0.143 criterion. RELION 2.1 was used to calculate the local resolution map [34].

Detailed workflows of image processing and statistics are shown in **S2–S4 Figs** and **S1 Table**.

### Model building and structure refinement

The crystal structure of the ClyA pore dodecamer (PDB accession code: 2WCD) [3] was used for the model building of the dodecamer against the electron microscopy density map and manually adjusted in COOT [39]. The new model was used for the model building of the tridecamer and the tetradecamer. Structure refinements were performed using PHENIX [40] in real space with secondary structure and geometry restrained. Model validations were performed using previously described methods to avoid overfitting [41, 42]. The statistics of the geometries of the models were generated using MolProbity [43].

All structure figures were prepared with PyMol [44] and all EM density map figures were generated with Chimera [45].

### Fluorescence spectroscopy

Fluorescence emission spectra of ClyA were monitored with a PTI QuantaMaster Fluorometer. The emission spectra from 300 nm to 400 nm were recorded at the excitation wavelength of 280 nm. Before recording the emission spectra, 5 μM ClyA protein (monomer) was incubated at RT for more than 2 h. The emission spectrum of each corresponding buffer was subtracted from the ClyA-containing sample. The highest reading was set as 100% for normalization.

### Liposome leakage assay

Liposomes were prepared as described previously with some modifications [18]. Briefly, lipids were dried in glass vials under a stream of air and placed at RT (~ 24°C) for more than 2 h. The lipid film was resuspended with 300 μL of buffer, containing 10 mM HEPES, pH 7.4, 25 mM NaCl and 100 mM 5(6)-Carboxyfluorescein (CF) with around 300 mM NaOH for pH adjustment and solubilization of CF, to make a lipid suspension of POPC-DOPS (85%:15%) at 15 mM or porcine brain total lipid extract (BTLE) at 15 mg/mL (Avanti Polar Lipids, Inc). The lipid suspension was vortexed continuously for 5 min and then subjected to at least five freezing-thawing cycles with liquid nitrogen. To obtain uniform liposomes, the lipid suspension was extruded for at least 31 times through a 0.1 μm membrane filter (Whatman) using an Avanti Mini Extruder device (insoluble precipitates in BTLE suspension were removed by centrifugation before extrusion). Liposomes were separated from unincorporated CF dyes using PD-10 desalting columns (GE Healthcare) in the elution buffer containing 10 mM HEPES, pH 7.4, and 150 mM NaCl.

The leakage assay was performed at RT and started when liposomes were added into the assay buffer (50 mM MES, pH 5.5, or 50 mM HEPES, pH 7.4, and 150 mM NaCl) to make a starting solution of 90 μL, corresponding to around 0.3 mM of lipid in the volume of 100 μL. At 160 s, 10 μL of the assay buffer or protein was added, resulting in a protein concentration of 0.5 μM. At 600 s, 10 μL of 10% n-Octyl-β-D-Glucopyranoside (β-OG) was added for liposome disruption and full release of CF. The assay was ended at 800 s. During the whole process, the fluorescence signal was monitored by the same fluorometer as for the fluorescence spectroscopy, with λ_Ex_ at 480 nm and λ_Em_ at 517 nm. Fluorescence readings were calculated as the percentage of CF dye release, with the reading at 160 s set as 0% and the reading at 700 s set as 100%.

For negative staining examination of liposome samples, the systems were prepared similarly as leakage assay, but the liposomes were not disrupted by β-OG.

## Supporting information

S1 Fig Assembly of the ClyA pore complexes. (**A**) Size exclusion chromatography (SEC) profile of ClyA as soluble protein and pore complex. The elution volumes were 15.2 mL and 9.8 mL, respectively. The column used here was Superdex 200 10/300 GL (GE Healthcare). (**B**) Negative staining images of ClyA in the form of soluble protein or pore complex. Fractions indicated in (**A**) were applied to negative staining (“-” indicates the soluble protein, “+1” or “+2” indicates the pore complexes). (**C**) Different strategies for preparing ClyA pore complex samples. Detailed data analysis of the samples is found in **S2**–**S4 Figs**.
(TIF)

S2 Fig Data analysis of the ClyA pore complex micrographs obtained on Talos Arctica. (**A, B**) Representative 2D class averages of the ClyA pore complexes in DDM (sample #1) or in digitonin (sample #2). Top view (or bottom view, not differentiable) of the dodecamer or the tridecamer is indicated. (**C, D**) Flowchart of data processing with sample #1 or sample #2.
(TIF)

S3 Fig Flowchart of data processing with micrographs obtained on Titan Krios. Sample #3 was used for data collection with Titan Krios. More details are described in materials and methods and shown in **S4 Fig**.
(TIF)

S4 Fig Analysis of cryo-EM data obtained on Titan Krios. (**A**) A representative electron micrograph. Three particles are indicated by the red arrow or red circle (top view) and magenta circle (side view). (**B**) Representative 2D class averages. Top view images showing 12, 13 or 14 symmetric features are indicated. (**C**) Gold-standard FSC curves for EM density maps of the dodecamer, the tridecamer, and the tetradecamer. (**D**) The angular distribution of particles used in the final 3D reconstruction, with the heights of the cylinders corresponding to the numbers of particles. (**E**) EM density maps colored by local resolution. (**F**) FSC curves for cross-validation between the models and the maps. Curves for the final refined model versus the reconstruction from all particles in black (sum), for the model refined against the reconstruction from only half of the particles versus the same reconstruction in blue (work), and for the same model versus the reconstruction from the other half of the particles in red (free).
(TIF)

S5 Fig Local density maps for the ClyA pore complexes. Representative residues are labeled for comparison between different maps. (**A**) The electron density maps for the crystal structure of the ClyA pore complex (PDB accession code: 2WCD) [3]. The maps, shown as blue mesh, are contoured at 1.0 σ. (**B-D**) The EM density maps for the dodecamer, the tridecamer, and the tetradecamer of the ClyA pore complexes, which are contoured at 5.0 σ and shown as blue meshes.
(TIF)

S6 Fig The electrostatic surface potential of the ClyA pore complexes. Side and bottom views of the dodecamer, the tridecamer, and the tetradecamer are shown. The figures were generated in PyMol, shown at the default contour level (~ +/- 70 *k*_B_T/e).
(TIF)

S7 Fig The interaction between 2 neighboring protomers in the crystal structure of the ClyA pore complex. The figure is presented in the same way as **Fig 2D** and **2E**.
(TIF)

S8 Fig A potential intermediate state of ClyA. (**A**) Representative cryo-EM 2D class averages of the ClyA intermediate state. (**B**) 3D reconstruction cryo-EM density map of the ClyA trimer at 16 Å. (**C**) The crystal structure of the ClyA monomer (PDB accession code: 1QOY) [12] and the structure of one protomer from the dodecamer of the ClyA pore complex (Prot_12) [3]. In the monomer structure, elements including αA1, αA2, αD, β-tongue and αE, which undergo significant conformational changes and insert into the membrane, are colored and labeled. The scaffold elements, including αB, αC, αF and αG, are colored grey. The corresponding elements are colored the same for consistency in the structure of Prot_12. (**D**) Fluorescence emission spectra of the ClyA monomer and the pore complexes, with or without the purification tag. Wavelengths of 316, 323, and 330 nm are indicated for visualization of peak shift. Curves for ClyA and ClyA-pore (DDM) are identical to those used in **Fig 3D**. (**E**) Raw recording data for **Fig 3D** and **S8D**.
(TIF)

S9 Fig Liposome leakage induced by ClyA. (**A**) POPC-DOPS liposomes were incubated with tagged ClyA for leakage test at pH 5.5 or 7.4. 100 mM CF was incorporated into liposomes and the release of CF resulted in an increase of fluorescence signal due to dequenching. Buffer and VP1682 were tested as negative controls. VopQ was adopted as the positive control. (**B**) Leakage of BTLE liposomes caused by ClyA, but not ClyA pore complexes (pH 7.4). (**C**) Negative staining images of POPC-DOPS liposomes incubated with 0.5 μM ClyA at RT for 40 min (pH 5.5).
(TIF)

S1 Table Cryo-EM data and structure model statistics. (PDF)
