# Supplementary figures and images for: High-resolution cryo-EM structures of the E. coli hemolysin ClyA oligomers
Source: PLoS One. 2019 May 2;14(5):e0213423. doi: 10.1371/journal.pone.0213423 (PMC6497250; doi:10.1371/journal.pone.0213423)

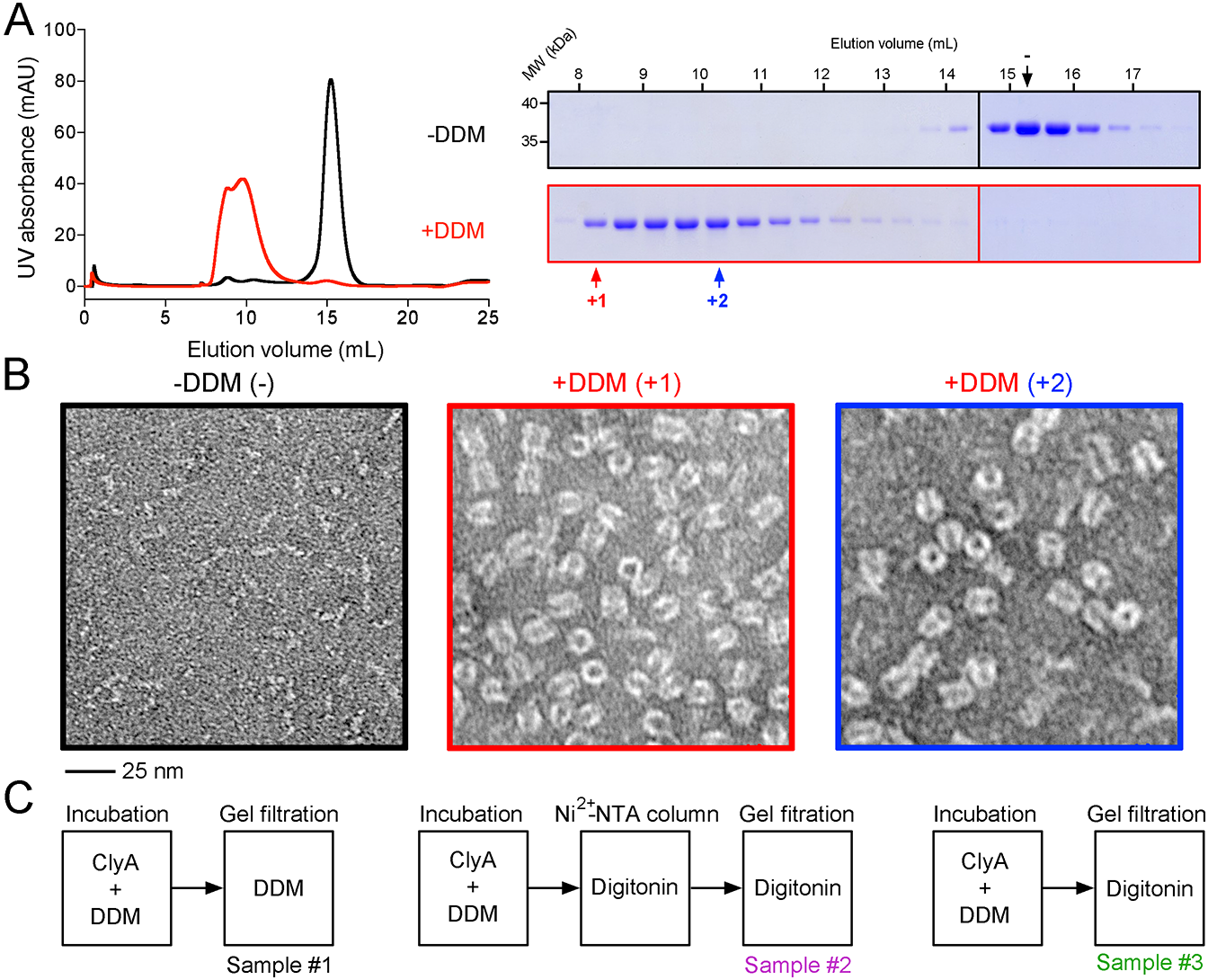

Supplement: S1 Fig — (A) Size exclusion chromatography (SEC) profile of ClyA as soluble protein and pore complex. The elution volumes were 15.2 mL and 9.8 mL, respectively. The column used here was Superdex 200 10/300 GL (GE Healthcare). (B) Negative staining images of ClyA in the form of soluble protein or pore complex. Fractions indicated in (A) were applied to negative staining (“-” indicates the soluble protein, “+1” or “+2” indicates the pore complexes). (C) Different strategies for preparing ClyA pore complex samples. Detailed data analysis of the samples is found in S2–S4 Figs. (TIF) [file pone.0213423.s001.tif]

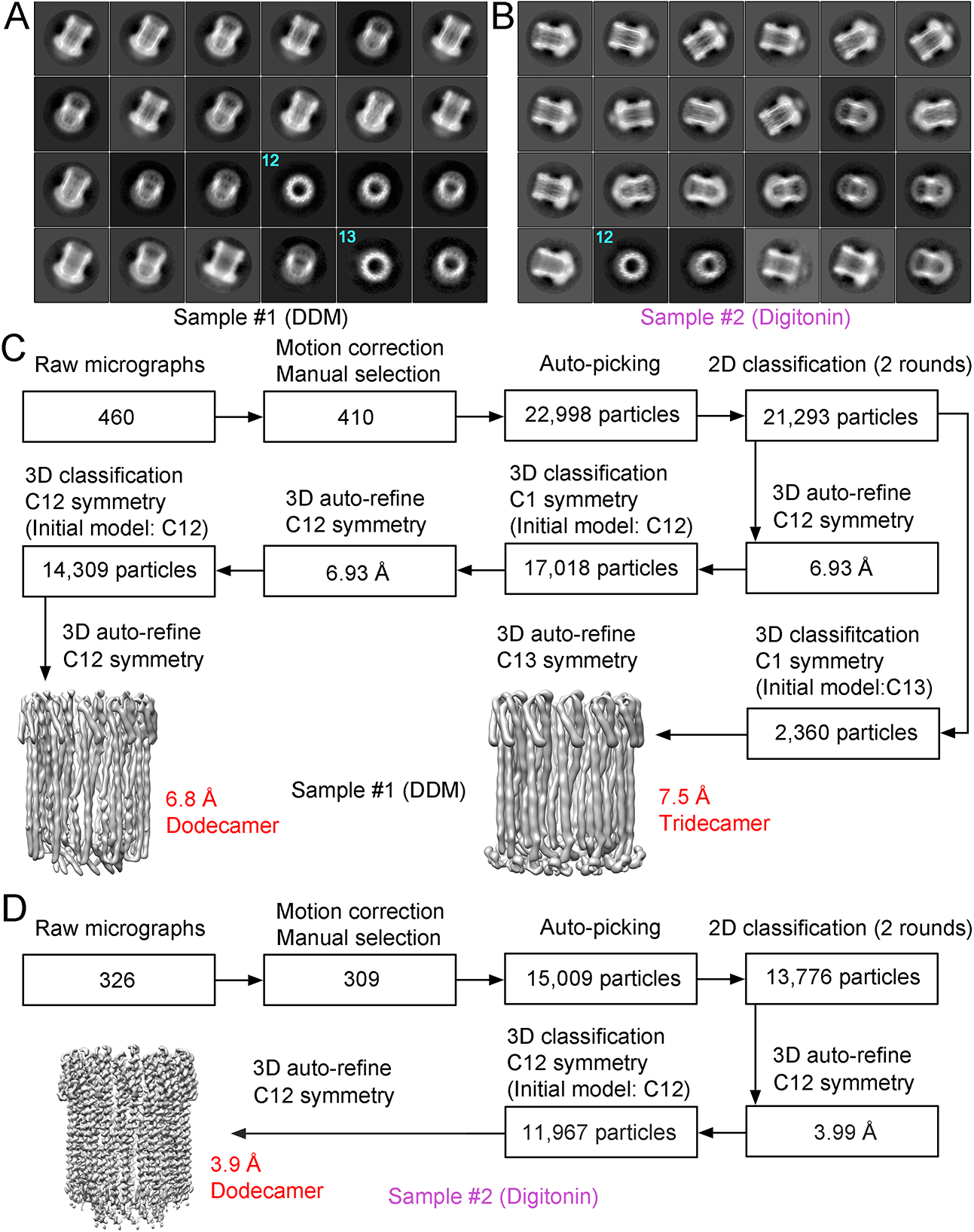

Supplement: S2 Fig — (A, B) Representative 2D class averages of the ClyA pore complexes in DDM (sample #1) or in digitonin (sample #2). Top view (or bottom view, not differentiable) of the dodecamer or the tridecamer is indicated. (C, D) Flowchart of data processing with sample #1 or sample #2. (TIF) [file pone.0213423.s002.tif]

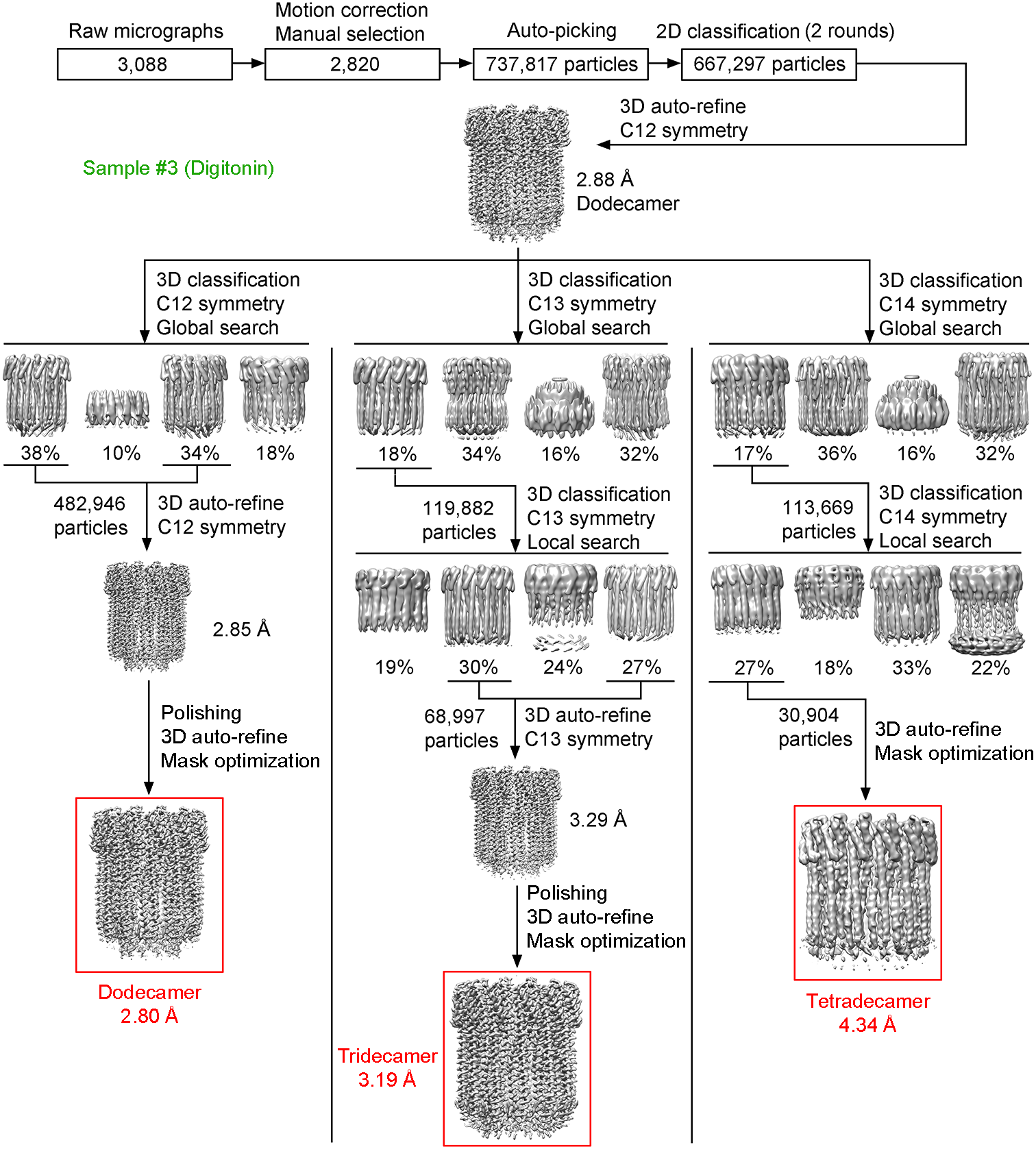

Supplement: S3 Fig — Sample #3 was used for data collection with Titan Krios. More details are described in materials and methods and shown in S4 Fig. (TIF) [file pone.0213423.s003.tif]

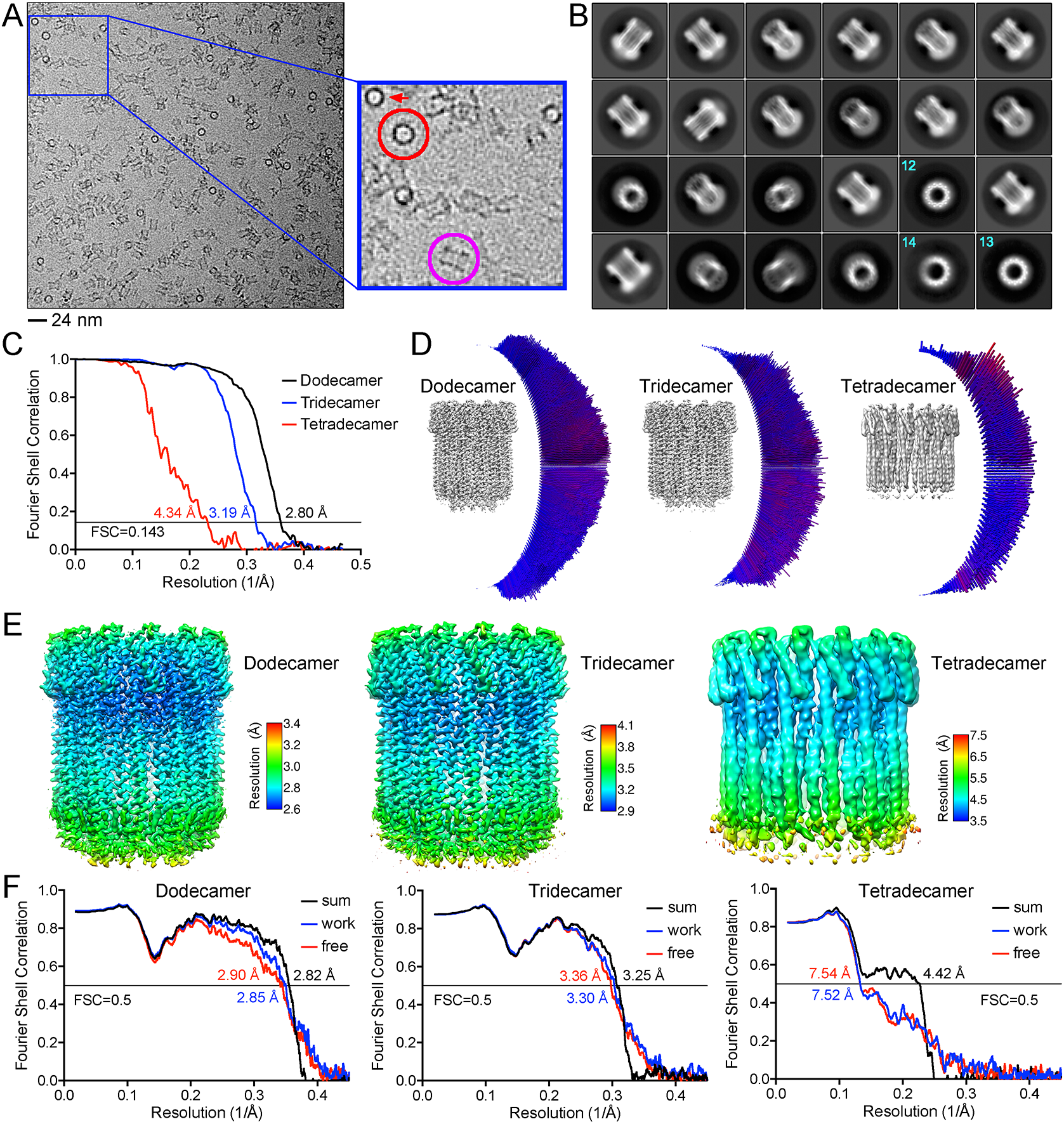

Supplement: S4 Fig — (A) A representative electron micrograph. Three particles are indicated by the red arrow or red circle (top view) and magenta circle (side view). (B) Representative 2D class averages. Top view images showing 12, 13 or 14 symmetric features are indicated. (C) Gold-standard FSC curves for EM density maps of the dodecamer, the tridecamer, and the tetradecamer. (D) The angular distribution of particles used in the final 3D reconstruction, with the heights of the cylinders corresponding to the numbers of particles. (E) EM density maps colored by local resolution. (F) FSC curves for cross-validation between the models and the maps. Curves for the final refined model versus the reconstruction from all particles in black (sum), for the model refined against the reconstruction from only half of the particles versus the same reconstruction in blue (work), and for the same model versus the reconstruction from the other half of the particles in red (free). (TIF) [file pone.0213423.s004.tif]

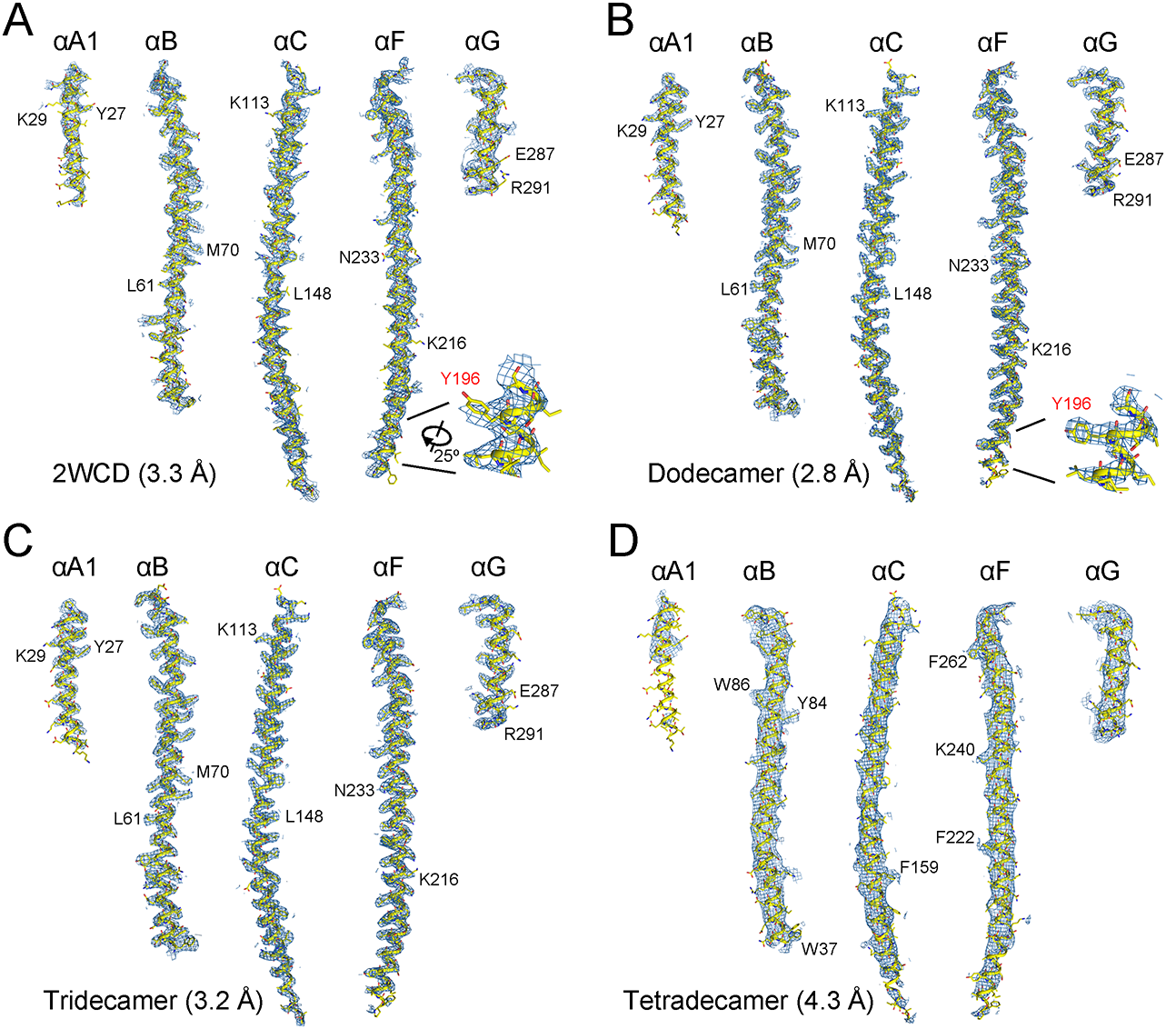

Supplement: S5 Fig — Representative residues are labeled for comparison between different maps. (A) The electron density maps for the crystal structure of the ClyA pore complex (PDB accession code: 2WCD) [3]. The maps, shown as blue mesh, are contoured at 1.0 σ. (B-D) The EM density maps for the dodecamer, the tridecamer, and the tetradecamer of the ClyA pore complexes, which are contoured at 5.0 σ and shown as blue meshes. (TIF) [file pone.0213423.s005.tif]

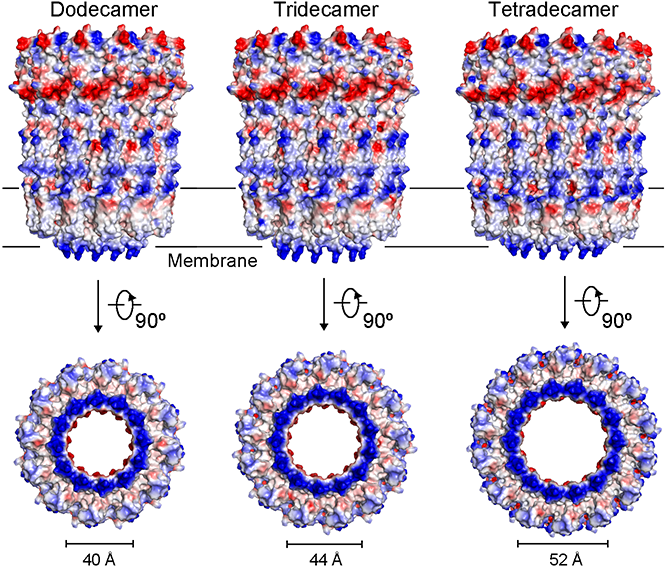

Supplement: S6 Fig — Side and bottom views of the dodecamer, the tridecamer, and the tetradecamer are shown. The figures were generated in PyMol, shown at the default contour level (~ +/- 70 kBT/e). (TIF) [file pone.0213423.s006.tif]

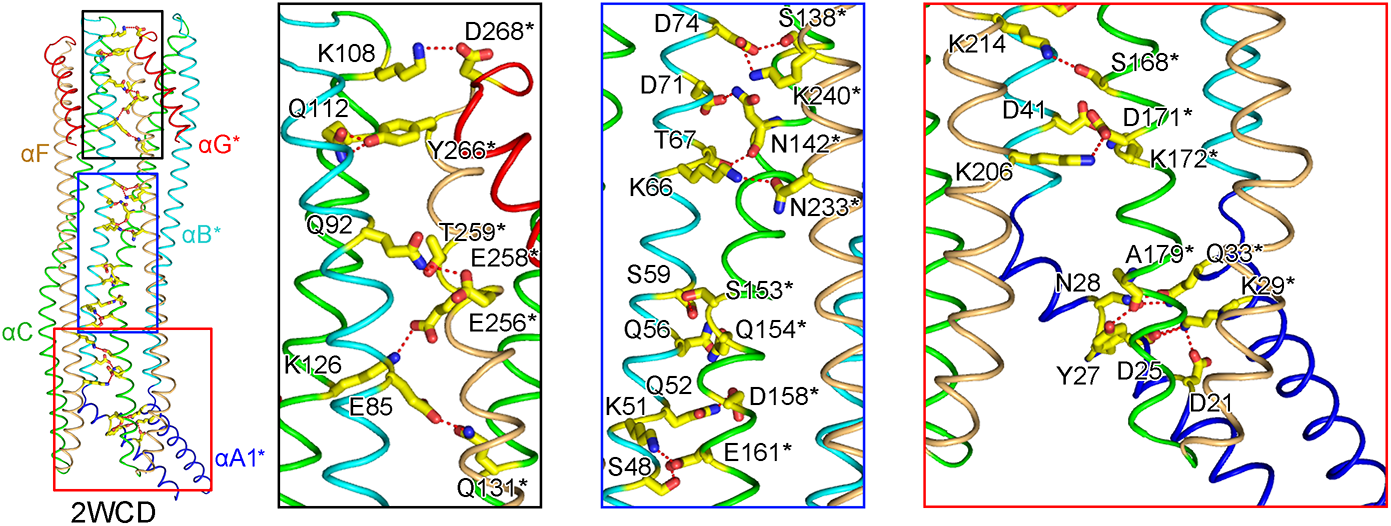

Supplement: S7 Fig — The figure is presented in the same way as Fig 2D and 2E. (TIF) [file pone.0213423.s007.tif]

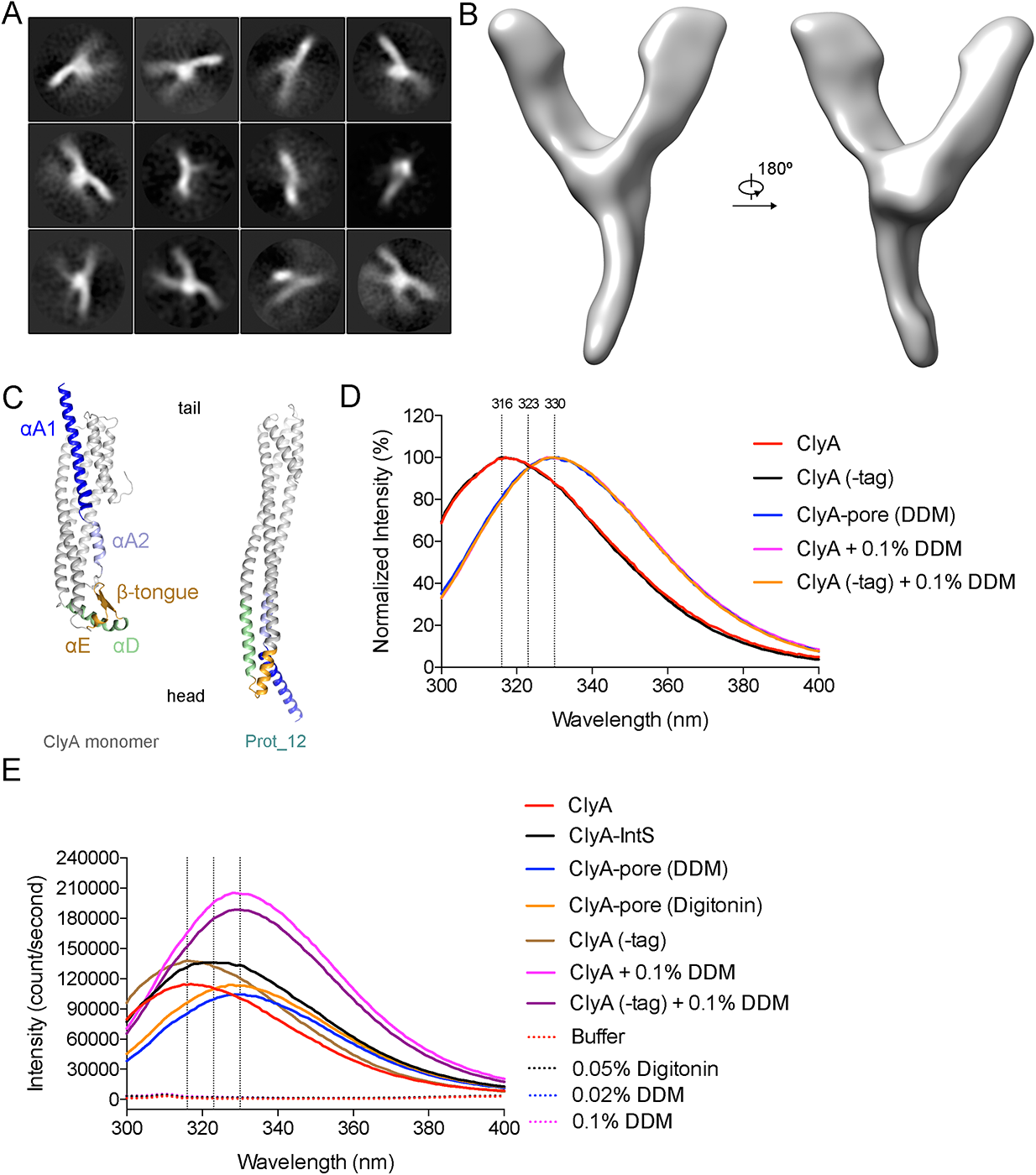

Supplement: S8 Fig — (A) Representative cryo-EM 2D class averages of the ClyA intermediate state. (B) 3D reconstruction cryo-EM density map of the ClyA trimer at 16 Å. (C) The crystal structure of the ClyA monomer (PDB accession code: 1QOY) [12] and the structure of one protomer from the dodecamer of the ClyA pore complex (Prot_12) [3]. In the monomer structure, elements including αA1, αA2, αD, β-tongue and αE, which undergo significant conformational changes and insert into the membrane, are colored and labeled. The scaffold elements, including αB, αC, αF and αG, are colored grey. The corresponding elements are colored the same for consistency in the structure of Prot_12. (D) Fluorescence emission spectra of the ClyA monomer and the pore complexes, with or without the purification tag. Wavelengths of 316, 323, and 330 nm are indicated for visualization of peak shift. Curves for ClyA and ClyA-pore (DDM) are identical to those used in Fig 3D. (E) Raw recording data for Fig 3D and S8D. (TIF) [file pone.0213423.s008.tif]

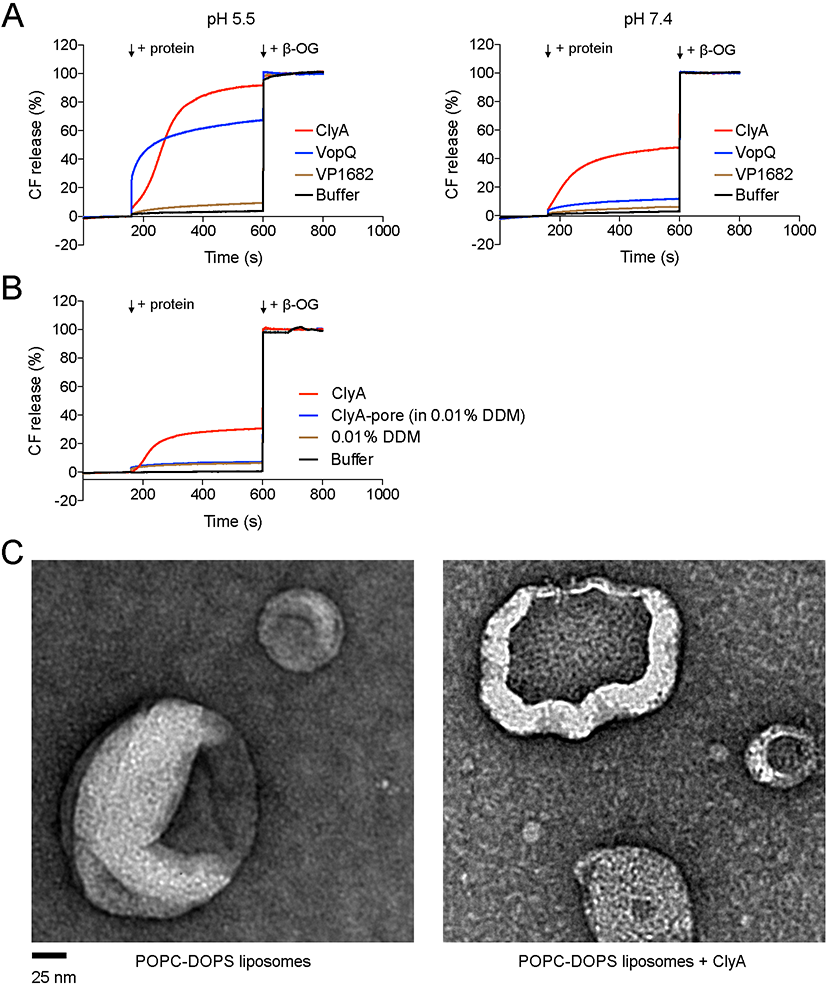

Supplement: S9 Fig — (A) POPC-DOPS liposomes were incubated with tagged ClyA for leakage test at pH 5.5 or 7.4. 100 mM CF was incorporated into liposomes and the release of CF resulted in an increase of fluorescence signal due to dequenching. Buffer and VP1682 were tested as negative controls. VopQ was adopted as the positive control. (B) Leakage of BTLE liposomes caused by ClyA, but not ClyA pore complexes (pH 7.4). (C) Negative staining images of POPC-DOPS liposomes incubated with 0.5 μM ClyA at RT for 40 min (pH 5.5). (TIF) [file pone.0213423.s009.tif]
