# Supplementary material for: High-resolution cryo-EM structures of the E. coli hemolysin ClyA oligomers
Source: PLoS One. 2019 May 2;14(5):e0213423. doi: 10.1371/journal.pone.0213423 (PMC6497250; doi:10.1371/journal.pone.0213423)
Supplement: S1 Table — (PDF) [file pone.0213423.s010.pdf]

	Dodecamer	Tridecamer	Tetradecamer
**Data collection and processing**			
Voltage (kV)		300	
Magnification		46,730	
Pixel size (Å)		1.07	
Electron dose ($e^-/Å^2$)		~ 50	
Defocus range (μm)		-1.6 ~ -2.6	
Final particle number	482,946	68,997	30,904
Symmetry	C12	C13	C14
Final Resolution (Å)	2.80	3.19	4.34
Map sharpening B-factor ($Å^2$)	-122.1	-118.6	-191.9
**Model building & Refinement**			
Protein residues	285 × 12	285 × 13	285 × 14
B-factors	43.38	52.99	208.88
R-factor	0.324	0.333	0.355
RMS deviation			
Bonds length (Å)	0.008	0.005	0.006
Bonds Angle (º)	0.896	0.723	0.899
Molprobity statistics			
Ramachandran plot (%)			
Outliers	0.00	0.00	0.00
Allowed	1.06	1.41	2.12
Favored	98.94	98.59	97.88
Rotamer outliers (%)	0.82	0.82	0.41
Clashscore	1.61	2.27	4.45
Overall score	0.91	1.00	1.25
